# Risk of nephrotoxicity among dry cleaning workers exposed to perchloroethylene: A comparative cross-sectional study in Addis Ababa, Ethiopia

**DOI:** 10.1371/journal.pone.0348427

**Published:** 2026-04-30

**Authors:** Philemon Mohammed Seid, Wossen Habtu, Meaza Gezu Shentema, Teferi Abegaz

**Affiliations:** 1 Department of Occupational Health and Safety, Wollo University, College of Medicine and Health Science, Dessie, Ethiopia; 2 Department of Preventive Medicine, Addis Ababa University, School of Public Health, Addis Ababa, Ethiopia; 3 Department of Clinical Chemistry, Ethiopian Public Health Institute, Addis Ababa, Ethiopia; Universiti Tunku Abdul Rahman Fakulti Perubatan dan Sains Kesihatan M Kandiah, MALAYSIA

## Abstract

Perchloroethylene, a chemical commonly used in the dry-cleaning sector, presents a potential risk to kidney health. This study measured urinary and blood nephrotoxic biomarkers among dry cleaners compared to hotel laundry workers to assess PCE’s impact. A multi-center comparative cross-sectional study was conducted with 164 randomly selected participants from 21 dry-cleaning shops and 26 hotel laundries. Data were collected through biological samples and structured questionnaires and with tests for statistical significance. The result showed that majority of the employees were females in both groups and the risk of nephrotoxicity was higher in dry cleaners as compared to hotel laundry workers. We found a significant mean difference in three biomarkers namely, Total Protein (TPU) with a Median & IQR value of (102 mg/dl &70.75 mg/dl) and (54.5 mg/dl &27.25 mg/dl), Urinary creatinine with a Median & IQR value of (193 mg/dl & 111.06 mg/dl) and (142.93 mg/dl & 78.17 mg/dl) and Urinary Calcium with a Median & IQR value of (2.60 mmol/l & 2.94 mmol/l) and (0.835 mmol/l & 0.79 mmol/l) for the exposed and the control groups respectively. However, a significant difference was not found in urinary protein to creatinine ratio, urinary sodium, Blood urea nitrogen and S. creatinine between the two groups, but higher value of sodium above range and higher BUN within range was observed in dry cleaners and factors like employment duration, PCE spillage, handling frequency, sex, and poor ventilation correlated with immediate symptoms. In conclusion, dry cleaners are at greater risk of kidney damage linked to PCE exposure, warranting implementation of safety measures and regular health monitoring to protect workers.

## Introduction

Perchloroethylene (PCE), also known as tetrachloroethylene, which is a colorless, non-flammable liquid solvent primarily used in the dry cleaning industry, has been known to cause significant public health problems [1]. The application of PCE as a cleaning solvent started in the 20th century [2]. It is also an important metal-degreasing solvent and an intermediate for other chemical production [3]. It is slightly soluble in water, having a boiling point of 121.4 °C, and a higher density than air, which makes good ventilation in an occupational setting mandatory [2].

Dry cleaners are the most occupationally exposed group to PCE [4]. Inhalation is the major route of entry [5] and It takes 3 days for half of it to be removed from a human body [1]. PCE has been identified by the International Agency for Research on Cancer as a probable carcinogen, with a very limited study on humans [6]. It has both acute and chronic health impacts, causing many health issues affecting the nervous system, kidneys, and liver [1]. It is believed to be a nephrotoxic, skin, and respiratory irritant [4].

During the 1990s, the use of perchloroethylene (PCE) increased rapidly, particularly in dry-cleaning shops where it became a commonly used solvent. This trend drew the attention of public health researchers, prompting efforts to assess occupational exposure. Consequently, numerous exposure assessment studies were conducted during this period [6,7]. Some health effect studies have been conducted in Western and Asian countries suggesting a link between perchloroethylene (PCE) exposure and nephrotoxicity [7–9]. Kidney toxicity, including both acute kidney injury (AKI) and chronic kidney disease (CKD), is a major global health concern, affecting an estimated 10% of the world’s population [10]. Research indicates that workers exposed to perchloroethylene (PCE), such as those in the dry-cleaning industry, are at increased risk of developing kidney-related health problems [5]. Unfortunately, consistent and conclusive evidence linking PCE exposure to kidney toxicity remains limited.

The American Conference of Governmental and Industrial Hygienists (ACGIH) suggests conducting further research regarding the health effects of PCE [7]. A study conducted in Italy also highlights the need to do research on the health effects of PCE at even low occupational doses [11]. The metabolism of PCE in the human body takes two pathways [12]. The first one is oxidative metabolism, which involves P_450_ to convert it into TCA(trichloroacetic acid) and others, causing liver and kidney toxicity [13]. The second metabolism is GSH (glutathione conjugation), which results in nephrotoxic metabolites and causes acute proximal tubal injury and kidney damage [14]. but the effect of GSH metabolites on kidney toxicity was not assured, but rather hypothesized [12].

Kidney toxicity is one of the critical concerns of PCE health effects [5]. Chronic exposure to it has been associated with end-stage renal disease [8]. Few studies have tried to study the renal effects of PCE among dry cleaners and found inconsistent results [1]. Since the practice of dry cleaning is increasing in Ethiopia, it is crucial to understand the extent of its health impacts among dry cleaners to adopt and implement safer working practices.

Biomarkers are essential in understanding the nephrotoxic effects of PCE. Proteinuria serves as a key biomarker for detecting PCE-induced nephrotoxicity. Some studies have shown elevated total protein (TPU), indicating glomerular injury from PCE toxicity in both animals and humans [15,16]. However, other research has found no significant or consistent association, highlighting inconsistent evidence on this effect [17].

According to a case report in South Korea, urinary calcium levels are elevated due to calcium crystals in the renal tubules caused by PCE exposure [18]. Similar to this, low urinary sodium excretion often precedes the diagnosis of AKI, especially in critically ill patients [19]. BUN and S. creatinine, which are standard kidney function biomarkers [20], would show impaired urea excretion and reduced GFR, respectively, as a result of PCE exposure toxicity [15,16,19].

Despite acknowledging limitations in our search coverage, we found no notable studies on PCE exposure and its health effects in Africa or Ethiopia. This highlights a significant knowledge gap, especially as the dry-cleaning sector continues to grow in the region. In Ethiopia, the increasing number of dry-cleaning shops has raised concerns about occupational exposure to perchloroethylene (PCE), making it an emerging public health issue. This study investigated the relationship between perchloroethylene (PCE) exposure and nephrotoxicity in Ethiopia, where no prior data on this association currently exists, and tried to fill the existing knowledge gap and provide evidence-based recommendations for improving occupational health standards in the Ethiopian dry-cleaning industry.

## Methods

A multicenter comparative cross-sectional study was conducted in Addis Ababa from March 8, 2025, to April 7, 2025. Using simple random sampling from the “addisbiz” local directory, 17 of 62 dry-cleaning shops were chosen by lottery, enrolling 70 dry cleaners. Similarly, 21 of 547 hotels were randomly selected to recruit 70 non-exposed controls. For blood sampling, an additional 4 dry-cleaning shops and 5 hotels (from unselected establishments) were randomly picked, with 12 participants obtained from each group.

Hospitality laundry workers were selected as the control group because they perform physically and environmentally comparable tasks such as handling, washing, ironing, and processing textiles in similar hot, humid workplace conditions with exposure to water-based detergents and mechanical stressors, yet without routine use of perchloroethylene solvents. We have selected only those hotels that doesn’t provide dry-cleaning service. This design helps isolate the potential nephrotoxic effects of PCE while minimizing confounding from shared socioeconomic factors, occupational physical demands, shift patterns, and general service-sector exposures common in low- to middle-income settings like Addis Ababa. Alternative control populations, such as office workers or the general community, were considered but deemed less suitable due to substantial differences in physical workload, heat/humidity exposure, and socioeconomic status, which could introduce bias in biomarker comparisons related to kidney function. Similar control selection strategies (e.g., using non-solvent-exposed laundry workers) have been employed in prior occupational studies of PCE-exposed dry cleaners to enhance comparability and internal validity.

### Sample size calculation

The sample size was calculated using the formula for comparative cross-sectional studies. However, because we could not identify definitive values for the effect size, mean, or standard deviation for both groups and the available data only pertained to workers’ exposure status, which would have greatly limited our sample size for assessing nephrotoxicity, we opted to use Cohen’s d. We assumed a medium effect size of 0.5, which is commonly considered appropriate for detecting meaningful differences in public health studies with a 1:1 allocation ratio


$$\text{n}=\frac{{\left(\frac{{\text{Z}}_{\alpha }}{\text{2}}+{\text{Z}}_{\beta }\right)}^{\text{2}}*\left(\overset{\text{2}}{\underset{\text{1}}{\sigma }}+\overset{\text{2}}{\underset{\text{2}}{\sigma }}\right)}{{\left({\text{M}}_{\text{1}}-{\text{M}}_{\text{2}}\right)}^{\text{2}}}$$

Cohen’s d is calculated as:


D =((M_1− M_2 ))/ (SD)

Assuming equal variances between the two groups, the sample size calculation was simplified by using a common standard deviation (SD). To account for an anticipated 10% dropout rate, the initial sample size (n = 63 per group) was adjusted using the formula: New sample size = n/ (1 − dropout rate). This resulted in a required sample size of 70 participants per group (63/ 0.9 ≈ 70), yielding a total of 140 participants included in the study.

Taking the mean and standard deviation values for serum creatinine from a health impact assessment conducted in Iran [21], the sample size was calculated using the following formula. Based on the computation, the initial sample size was 10 per group. After accounting for a 10% dropout rate, the final sample size was adjusted to 12 participants per group. Assuming a one-to-one allocation ratio, a total of 24 participants were included, comprising 12 dry cleaners and 12 hotel laundry workers.


$$n\;=\left\{(\text{1}.\text{9}\text{6}\;+\;\text{0}.\text{8}\text{4}{)}^{\text{2}}\cdot \left({\text{0}.\text{2}\text{2}}^{\text{2}}+\;{\text{0}.\text{0}\text{4}}^{\text{2}}\right)\right\}\left\{(\text{1}.\text{1}\;-\;\text{0}.\text{9}\text{2}{)}^{\text{2}}\right\}$$

Workers aged 18 and older, employed for at least six months in dry-cleaning or hospitality laundry services, and able to give informed consent were eligible for the exposed group. Exclusions included those with pre-existing kidney disease before employment, those on temporary medication for non-kidney illnesses, pregnant or lactating women, and women menstruating during data collection due to metabolite variation.

Data were collected using structured questionnaires with both open-ended and closed-ended questions and biological samples. Ethical approval was obtained from Addis Ababa University School of Public Health, IRB committee with reference number SPH/452/2025, and Addis Ababa Health Bureau Ethical clearance committee with reference number 2/080/17. The study was conducted in accordance with the ethical principles for medical research involving human subjects outlined in the Declaration of Helsinki. Participants were asked to sign informed consent before collecting urine and blood samples, and were briefed to leave if they wanted to at any part of the research process without any constrictions. After getting their consent, willing participants were guided by trained collectors to provide clean catch mid-stream spot urine samples during their working hours following Ethiopian Public Health Institute (EPHI) protocols to reduce contamination. Urine samples, labeled with identification codes, were analyzed for nephrotoxicity biomarkers: proteinuria, creatinine, calcium, and sodium. Blood samples from 24 participants were collected by trained health professionals on Wednesdays and Thursdays after shifts to measure BUN and serum creatinine, reflecting PCE exposure. Samples were kept at 2–8°C, transported in cold boxes, and stored in EPHI laboratory refrigerators for later analysis using automated urine biochemistry analyzers.

The laboratory analysis was performed at the ISO 15189:2012-accredited EPHI National Clinical Chemistry referral and Reference Laboratory. Urinary biomarkers for kidney function were quantified in all 140 participants, and serum biomarkers in 24 participants. Protein in urine/CSF was measured via a turbidimetric method using cobas analyzers (range: 40–2000 mg/L). Calcium levels were determined photometrically with cobas analyzers (serum range: 0.20–5.0 mmol/L; urine: 0.20–7.5 mmol/L). Sodium concentrations were measured by ion-selective electrode on cobas c 111 (range: 20–250 mmol/L). Urine/CSF samples for protein were centrifuged to avoid precipitates; calcium and sodium assays required separation from cells within 4 hours, avoiding silicone-gel tubes to prevent interference.

Interferences such as hemolysis, icterus, lipemia, and certain drugs were monitored and mitigated according to defined thresholds. BUN was measured enzymatically (urease/GLDH method) on cobas c 501/502 (2–100 mg/dL range), and serum creatinine was determined by kinetic Jaffé methods on cobas c 503/703, calibrated to IDMS standards (0.2–25 mg/dL). Serum samples were processed within 2 hours, stored at 2–8°C. Data were cross-checked and cleaned by the principal investigator before analysis.

## Result

The basic demographic characteristics of both the exposed and control group workers from the selected 17 and 21 dry cleaning establishments and hotels, respectively are shown in Table 1. The majority of workers in both laundries were females, covering 61.4% of study participants in dry cleaning and 65.7% in hotel laundry. Participants’ sex, job role, working days, hours, and years of employment are shown in Table 1 along with their behavioral characteristics of smoking and drinking.

**Table 1:** Demographic characteristics of the study participants (N = 140).

Category	Description	Dry Cleaning Laundry	Hospitality Laundry	p- value
Age	Mean ± SD	30.66 ± 6.255	27.93 ± 4.261	<0.016
Sex	Female	43 (61.4%)	46 (65.7%)	<0.598
	Male	27 (38.6%)	24 (34.3%)	
Education level	Bachelor’s Degree	9 (12.9%)	14 (20.0%)	<0.428
	Diploma/Certificate	22 (31.4%)	18 (25.7%)	
	Elementary	9 (12.9%)	5 (7.1%)	
	High School	30 (42.9%)	33 (47.1%)	
Years of employment	Less than 1 year	9 (12.9%)	12 (17.1%)	<0.091
	1 to 3 years	28 (40.0%)	16 (22.9%)	
	4 to 6 years	21 (30.0%)	28 (40.0%)	
	7 to 10 years	10 (14.3%)	7 (10.0%)	
	11 to 15 years	2 (2.9%)	7 (10.0%)	
Job Role	Ironer	21 (30.0%)	14 (20.0%)	<0.546
	Machine Operator	25 (35.7%)	27 (38.6%)	
	Manager/Supervisor	11 (15.7%)	15 (21.4%)	
	Receptionist	13 (18.6%)	14 (20.0%)	
Working Days per Week	<3 days	1 (1.4%)	4 (5.7%)	<0.002
	4 days	3 (4.3%)	5 (7.1%)	
	5 days	9 (12.9%)	26 (37.1%)	
	6 days	55 (78.6%)	35 (50.0%)	
	7 days	2 (2.9%)	0 (0%)	
Work Hours per Day	4-6 hours	5 (7.1%)	11 (15.7%)	<0.298
	7-8 hours	53 (75.7%)	48 (68.6%)	
	>8 hours	12 (17.1%)	10 (14.3%)	
Smoking Status	Current Smoker	1 (1.4%)	4 (5.7%)	<0.392
	Former Smoker	4 (5.7%)	4 (5.7%)	
	Never Smoked	65 (92.9%)	62 (88.6%)	
Alcohol Consumption	No	19 (27.1%)	25 (35.7%)	<0.298
	Occasionally	45 (64.3%)	36 (51.4%)	
	Yes, frequently	6 (8.6%)	9 (12.9%)	
Family history of kidney disease	No	60(85.7%)	55(78.6%)	<0.028
	Yes	10(14.3%)	15(21.4%)	
BMI Category	Normal	43(61.4%)	58(82.9%)	<0.237
	Overweight	20(28.6%)	8(11.4%)	
	Obese	3(4.3%)	0	
	Underweight	4(5.7%)	4(5.7%0	
Other Health Conditions Affecting Kidney	Diabetes	1(1.4%)	4(5.7%)	<0.001
	Hypertension	0	5(7.1)	
	Liver Disease	1(1.4%)	1(1.4%)	
	None	68(97.1%)	60(85.7%)	

Comparison of key urinary biomarkers between dry cleaning workers (exposed group) and hospitality laundry workers (control group) is shown in Table 2. The data are expressed as median values with interquartile ranges (IQR) to illustrate the central tendency and variability within each group. This comparison highlights differences in total protein, creatinine, calcium, and sodium levels, providing insight into potential occupational exposure effects on renal function.

**Table 2:** Descriptive summary of the four urinary biomarkers between exposed and control groups (N = 140).

Variables	Exposed Group (Dry Cleaning workers)	Control Group (Hospitality laundry workers)
	Median & IQR	Median & IQR
Total Protein (mg/dl)	102.00 & 70.75	54.50 & 27.25
Urinary Creatinine (mg/dl)	193.78 & 111.06	142.93 & 78.17
Urinary Calcium (mmol/l)	2.60 & 2.94	0.835 & 0.79
Urinary Sodium (mmol/l)	167.00 & 67.00	158.00 & 73.75

Blood urea nitrogen (BUN) and serum creatinine levels between an exposed group and a control group, presenting means and standard deviations (SD) is presented in Table 3. The exposed group exhibits higher mean values for both biomarkers compared to the control group, with larger SDs indicating greater variability in the data. From the total of 12 samples in each group the prevalence of abnormal BUN was 33.3% (4 workers) and 16.7% (2 workers) in dry cleaners and hotel laundry workers respectively, while 3 workers in each group showed abnormal serum creatinine level with a prevalence of 25%.

**Table 3:** Descriptive summary of serum biomarkers comparing means of exposed and control groups (N = 24).

Variables	Exposed Group (Dry Cleaning) Mean ± SD	Control Group (Hospitality) Mean ± SD
BUN (mg/dl)	15.5 ± 6.08	12.5 ± 4.15
S. Creatinine (mg/dl)	0.8658 ± 0.36694	0.8262 ± 0.28804

The dry cleaning group reports higher percentages of decreased urine output, foamy/discolored urine, and dizziness/headache, while the hotel laundry group shows a higher prevalence of no changes in urination and no training on PCE handling. Fatigue/weakness and nausea/loss of appetite frequencies are relatively similar between groups, with slight variations in specific symptom prevalence. Health symptoms and training experiences between the two groups is presented below in Table 4.

**Table 4:** Disease symptoms and changes noticed in both dry cleaners and hotel laundry workers (N = 140).

Category	Description	Dry cleaning laundry	Hotel laundry
Urination patterns	Decreased urine output	22 (31.4%)	8 (11.4%)
	Foamy/discolored urine	16 (22.9%)	12 (17.1%)
	Increased frequency	5 (7.1%)	3 (4.3%)
	No changes	27 (38.6%)	47 (67.1%)
Fatigue/weakness	Always	13 (18.6%)	5 (7.1%)
	Never	7 (10.0%)	2 (2.9%)
	Often	31 (44.3%)	31 (44.3%)
	Sometimes	19 (27.1%)	32 (45.7%)
Nausea/loss of Appetite	Neither	29 (41.4%)	33 (47.1%)
	Both symptoms	13 (18.6%)	8 (11.4%)
	Loss of appetite only	14 (20.0%)	18 (25.7%)
	Nausea only	14 (20.0%)	11 (15.7%)
Training on PCE handling	No training	33 (47.1%)	53 (75.7%)
	Trained once	35 (50.0%)	15 (21.4%)
	Regularly trained	2 (2.9%)	2 (2.9%)
Dizziness and headache	No	12 (17.1%)	24 (34.3%)
	Yes	58 (82.9%)	46 (65.7%)

An independent t-test was performed to see if there was a significant, meaningful difference in urinary and serum biomarkers distribution between the exposed and the control groups in Table 5. The result showed that Log_10_ transformed total protein, urinary creatinine and calcium had a significant association with p < 0.05 for all three.

**Table 5:** Independent t-Test result of the log-transformed urinary biomarkers and serum biomarkers between exposed and control groups.

Urinary Biomarker	Dry Cleaning Workers (Mean ± SD)	Hospitality Laundry Workers (Mean ± SD)	T	df	P
Log10Total Protein(TPU)	1.988 ± 0.275	1.760 ± 0.192	5.67	123.3	P < 0.001*
Log10 Creatinine	2.217 ± 0.254	2.082 ± 0.242	3.22	137.6	P < 0.002*
Log10 Calcium	0.374 ± 0.359	−0.114 ± 0.407	7.49	134.4	P < 0.001*
BUN	15.5 ± 6.08	12.5 ± 4.15	−1.412	22	P < 0.172
S. creatinine	0.8658 ± 0.36694	0.8262 ± 0.28804	−0.295	22	P < 0.771
UPCR (Urinary protein to creatinine ratio)	0.54 ± 0.3315	0.64 ± 0.2945	−1.992	136.109	P < 0.791

*Shows significance.

Non- parametric Mann-Whitney U test was also conducted to see if there was a significant meaningful urinary sodium distribution difference between the exposed and the control groups, and the result showed a non-significant distribution with a p value of 0.176, which makes the null hypothesis true.

The renal function of participants was evaluated by comparing the estimated Glomerular Filtration Rate (eGFR) across the two occupational groups. To ensure a standardized assessment, the eGFR was calculated for each participant using the 2021 CKD-EPI creatinine equation (race-neutral), which incorporates serum creatinine, age, and sex as variable. The Mann-Whitney U test was employed to determine if significant differences existed between the groups. This non-parametric approach compares the distribution of ranks by providing a robust evaluation of potential impairment associated with occupational chemical exposure as shown in Table 6.

**Table 6:** Comparison of Renal Function (eGFR) by Occupational Group.

Variable	Dry Cleaning (n = 12)	Hospitality (n = 12)	p-value
eGFR, Median (mL/min/1.73m²)	126.9	111.1	0.544
(IQR)	(85.6–132.7)	(100.3–126.1)	

A correlation analysis was performed to see which risk factors significantly influence each specific biomarker. Point-Biserial correlation was conducted between risk factors and log-transformed urinary biomarkers of kidney function, and the result showed the strength and direction of the significantly associated results as shown in Table 7.

**Table 7:** Point-Biserial correlation result between log10 transformed urinary biomarkers and risk factors.

Variable	Log10 Total Protein (r, p)	Log10 Creatinine (r, p)	Log10 Calcium (r, p)	Log10 Sodium (r, p)
PCE spillage	0.201, < **0.017**	0.220, **< 0.009**	NS	NS
Workplace	0.434, **< 0.001**	0.264, **< 0.002**	0.539, **< 0.001**	NS
Family kidney history	NS	−0.178, **< 0.035**	−0.166, **< 0.050**	NS
Individual kidney history	NS	−0.179, **< 0.034**	NS	NS
Age in years	NS	NS	0.224, **< 0.008**	NS
Previous job PCE exposure	NS	NS	0.267, **< 0.001**	NS

Spearman correlation analysis was conducted as shown in Table 8, for urinary sodium and for the three urinary biomarkers to see if there is an association with variables that have an ordinal nature.

**Table 8:** Spearman correlation result between ordinal risk factors and urinary biomarkers.

Variable	Log10 Total Protein (ρ, p)	Log10 Creatinine (ρ, p)	Log10 Calcium (ρ, p)	Log10 Sodium (ρ, p)
Days worked in a week	0.378, < 0.001	0.354, < 0.001	NS	NS
Total hours worked in a day	0.183, < 0.030	NS	NS	NS
How often participants handle PCE	0.324, < 0.001	0.183, < 0.031	0.318, < 0.001	NS
How long participants are exposed to PCE in a day	0.272, < 0.001	0.254, < 0.002	0.378, < 0.001	NS
Hand washing after PCE handling	−0.188, < 0.026	NS	NS	−0.164, < 0.054
Education level	−0.208, < 0.014	−0.170, < 0.045	NS	NS
Training received	−0.161, < 0.058	NS	−0.196, < 0.021	NS
Size of the laundry in m²	–	–	–	0.175, < 0.039
Experience of PCE spillage	–	–	–	0.214, < 0.011
Previous job PCE exposure	–	–	–	0.173, < 0.041

### Abnormal urinary biomarkers

**Fig 1:**
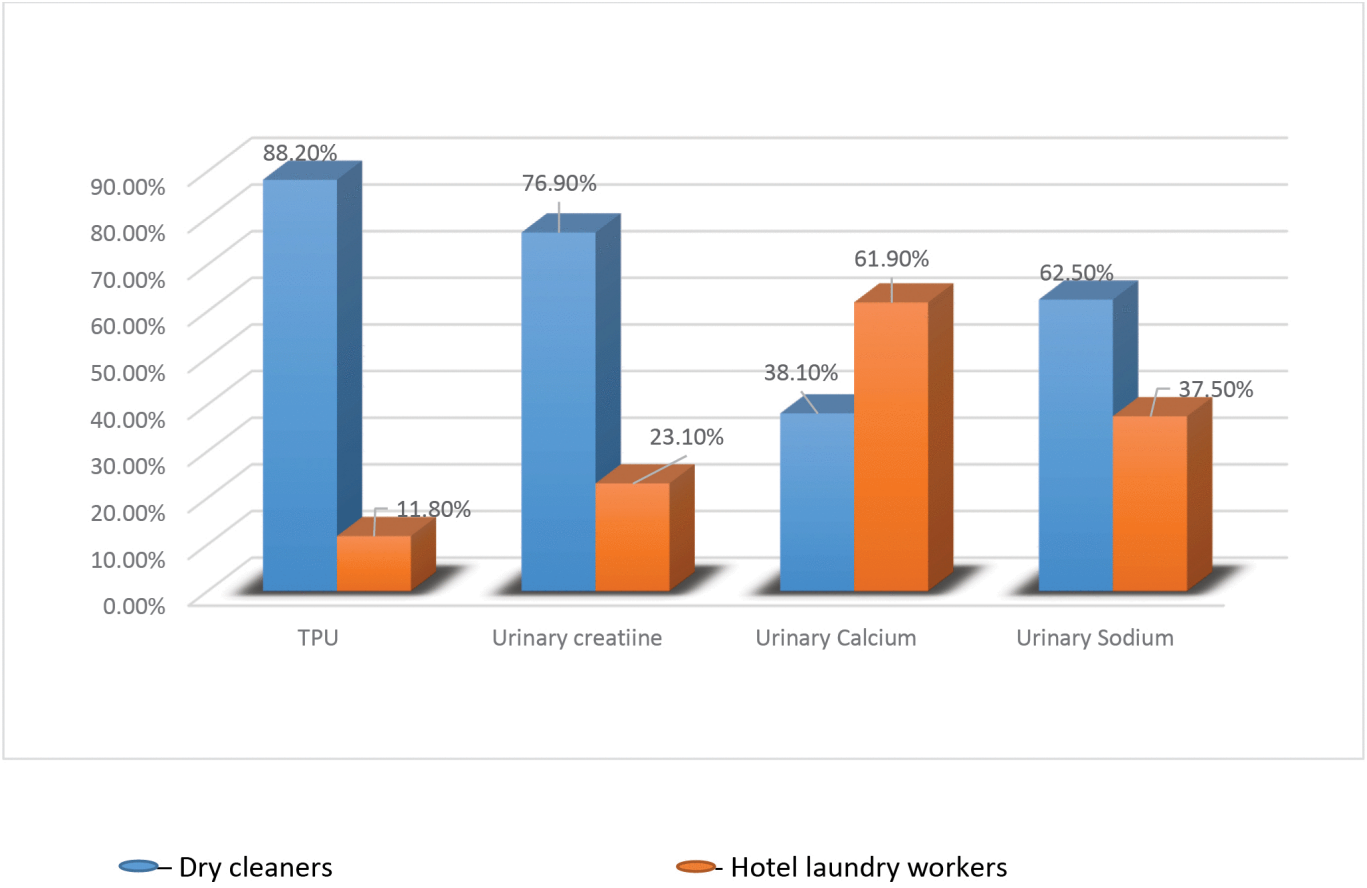
Clustered Bar graph comparing abnormal urinary biomarkers between dry cleaners and hotel laundry workers (N = 17 (TPU), N = 26 (Urinary creatinine), N = 97 (calcium), N = 8 (sodium)).

Total urinary protein less than 150 mg/dl is considered normal according to the EPHI guiding reference. From a total of 140 samples, 17 results showed deviation from the normal range, where most of the deviations are found in the dry-cleaning sector. 26 samples showed abnormal urinary creatinine results, where 29–226 mg/dl for females and 40–275 mg/dl for males were considered as a normal range. 97 tests showed abnormal urinary calcium results, where 2.5–7.5 mmol/l is considered normal. From the urinary sodium test, only 8 samples showed abnormal range, where 27–287 mmol/l for females and 40–220 mmol/l for males were considered in the normal range. The full comparison is shown in Fig 1 below.

A multiple linear regression analysis was conducted as shown in Table 9 to examine the effects of demographic characteristics, occupational factors, lifestyle characteristics, medical history, and workplace safety practices on urinary protein-to-creatinine ratio (UPCR). The model included age, days worked, type of ventilation, water intake, family history of kidney disease, personal history of kidney disease, chemical spillage, working hours, smoking, workplace, personal protective equipment use, hand washing practice, educational status, alcohol use, and health conditions like hypertension and diabetes.

**Table 9:** Multiple linear regression model showing significantly associated indicators for Urinary protein to creatinine ratio.

Variable	B	Std. Error	t	Sig.	95% Confidence Interval for B (Lower, Upper)	Tolerance	VIF
Constant	0.491	0.344	1.425	<0.157	(−0.191, 1.173)	—	—
Age in years	−0.018	0.006	−3.234	0 < .002	(−0.029, −0.007)	0.678	1.475
Workplace	0.209	0.076	2.751	<0.007	(0.059, 0.359)	0.443	2.255
Personal protective equipment use	−0.175	0.063	−2.769	<0.006	(−0.300, −0.050)	0.726	1.378
Alcohol use	0.096	0.047	2.034	<0.044	(0.003, 0.190)	0.756	1.323

The overall model was statistically significant (F (15,124) = 2.125, p = 0.013), explaining 20.4% of the variance in UPCR (R² = 0.204; adjusted R² = 0.108). A histogram of the residuals demonstrated an approximately normal distribution and the residual-versus-fitted plot showed a random scatter of points, indicating that the assumptions of normality and homoscedasticity were met. In addition, all predictors had tolerance values greater than 0.1 and variance inflation factor (VIF) values below 10, indicating absence of multicollinearity.

Increasing age was significantly associated with lower UPCR (B = −0.018, 95% CI: −0.029, −0.007; p = 0.002). Workplace category was positively associated with UPCR, with participants in the exposed workplace having significantly higher UPCR compared with the reference group (B = 0.209, 95% CI: 0.059, 0.359; p = 0.007). Use of personal protective equipment was significantly associated with lower UPCR (B = −0.175, 95% CI: −0.300, −0.050; p = 0.006). Alcohol use was also significantly associated with higher UPCR (B = 0.096, 95% CI: 0.003, 0.190; p = 0.044).

Other variables including days worked, type of ventilation, water intake, family history of kidney disease, personal history of kidney disease, chemical spillage, working hours, smoking status, hand washing practice, educational status, and general health condition were not significantly associated with UPCR in the adjusted model (p > 0.05).

A multiple linear regression analysis was also conducted in Table 10 below to examine the effects of PCE exposure predictors, demographic variables, health-related factors, and workplace safety practices on urinary total protein (mg/d). The model was statistically significant (*F* (14, 125) = 4.156, p < 0.001), and explaining 31.3% of the variance in proteinuria (R^2^ = 0.318 and adjusted R^2^ = 0.241). A histogram plot of the residuals showed a bell-shaped distribution, a residual verses fitted value plot showed a random scatter of points, all predictors Variance Inflation Factor (VIF) was below 10 and tolerance value for all factors were above 0.1, by which normality, homoscedasticity and absence of multi collinearity assumptions were met for the model.

**Table 10:** Multiple linear regression model showing significantly associated indicators for total protein.

Variable	B	Std. Error	T	Sig.	95% Confidence Interval for B (Lower, Upper)	Tolerance	VIF
Constant	1.945	0.281	6.946	<0.0001	(1.395, 2.507)		
Years of work	0.083	0.024	3.507	<0.001*	(0.036, 0.131)	0.568	1.761
Age in years	0.015	0.005	3.011	<0.003*	(−0.024, −0.005)	0.518	1.930
Work place	0.308	0.065	4.696	<0.001*	(0.178, 0.437)	0.348	2.872

A multiple linear regression was also conducted to see the effect of predictors to urinary creatinine level (mg/dl) after checking the assumptions in the same fashion done above for TPU. 10 predictors were included in the model, age, sex, BMI (Body Mass Index), work place, year of employment, working hour, water intake, smoking, alcohol consumption, history of kidney disease. The model was statistically significant (*F* (10, 129) = 2.412, p < 0.012), and explained 15.7% of the variance in urinary creatinine (R^2^ = 0.157 and adjusted R^2^ = 0.092).

Only two predictors were significant. The first one is a place of work, which had a significant positive effect (β = 0.152, p = 0.009), indicating that being a dry-cleaning worker is associated with 1.42 times increase in urinary creatinine, holding other variables constant. And the second one is years of employment (β = 0.051, p = 0.038), indicating that a one-year increase in laundry establishment results in 1.12 times increase in urinary creatinine, holding other variables constant.

Separate regression analysis was done for this two urinary biomarkers by adding approximated categorical dietary intake of sodium and calcium predictors from the recent meal they had before sample collection along with predictors like age, sex, BMI, place of work, working hour, water intake, smoking status, alcohol consumption, past history of kidney disease, experience of PCE spillage and availability of adequate ventilation. the model for urinary calcium was statistically significant (*F* (14, 124) = 4.21, p < 0.001), and explaining 32.9% of the variance in urinary calcium (R^2^ = 0.322 and adjusted R^2^ = 0.246), however the only significant predictor was place of work which had a significant positive effect (β = 0.438, p < 0.001), indicating being a dry cleaning worker is associated with a 2.74 times increase in urinary calcium, holding other variables constant. Regression analysis for urinary sodium showed a non-significant result in both the model and in all of the predictors, as a result, we excluded it from the regression report.

## Discussion

From the four urinary and two serum biomarkers, TPU was the most crucial and directly linked to Perchloroethylene induced nephrotoxicity [22]. Different studies have identified urinary protein levels as a sensitive indicator of kidney damage caused by toxic substances [23,24]. Our result showed a significant increase in proteinuria in dry cleaners as compared to hotel laundry workers, with a P-value of 0.001 in both parametric and non-parametric tests. Similar to our findings, studies conducted in Italy, Germany and the Netherlands to assess the renal effects of PCE in dry cleaners showed a significant increase in proteinuria in the exposed groups compared to the control groups [15,24,25]. Dry cleaners accounted for 88.2% of observed abnormal TPU (>150mg/d) in our study, while 87% of subjects in the Italy study showed an increased high molecular weight protein in urine, which was associated with tubular alteration [25]. On the other hand, some studies contradicted our finding. A study conducted in the US found no direct association between PCE exposure and urinary biomarkers from 192 dry cleaning workers, even though protein/creatinine showed a weak positive association [17]. In contrast to the previous studies, a study conducted in the Czech Republic found no significant difference in total urinary protein between the exposed (125 mg/g) and control group (103 mg/g), countering the general claim of a clear association between PCE exposure and proteinuria [16], Prevalence of abnormal value of protein showed no significance between the exposed and control groups in another study conducted in the Netherlands [26]. Whereas abnormal TPU in our findings showed a significant association by which hotel laundry workers had 88.2% lower odds of TPU abnormality compared to dry cleaners.

According to the agreed-upon pathophysiological study of Proteinuria, PCE exposure results in higher protein in urine by compromising the glomerulus, which is the key filtration unit within in nephron that allows the passage of tiny water and electrolyte molecules, while blocking large molecules like protein [27]. When this filtration unit is compromised by PCE (controlling for other factors like diabetes, hypertension), protein output in urine increases [12,28].

Urinary creatinine was used to show the effect of PCE on kidney filtration as a result of toxic-induced renal damage, resulting in GFR reduction [29]. It is a key indicator of overall kidney function, since it is a result of creatine degradation from muscle [30]. However, most of the time, it is used as a normalizer for other biomarkers [31]. PCE exposure is believed to decrease GFR [29], which in turn decreases urinary creatinine and indicates acute and chronic kidney disease [32].

Some studies used S. creatinine only as a biomarker to detect nephrotoxicity, but found no significant association, which is similar to our S. creatinine finding. The results of a study in Iran found no significant difference in Cr levels between dry-cleaners (1.1 mg/dl) and control group (0.92 mg/dl) [21]. Similarly, a study conducted in China to see the effects of the exposure on liver and kidney functions yielded negative results as judged by the emission enzyme activities [33]. Complimentarily a study in Italy found that chronic exposure to organic solvents did not significantly alter urinary creatinine level, although it stated that urinary creatinine can’t be used to detect renal dysfunction alone [34].

Our research found a significant urinary creatinine difference between the exposed and the control group, however majority of them showed an increased urinary creatinine above the range (>226 mg/dl for female and >275 mg/dl for males). Which might come from renal tubular damage caused by PCE metabolites like S-(1, 2, 2-trichlorovinyl)-L-cysteine (TCVC), which induce oxidative stress and affect tubular integrity [15,30]. As a result kidneys’ ability to filter will decrease, and GFR would fall, so to compensate for this, the proximal tubules would increase creatinine secretion and another possibility is tubular epithelial barrier might be compromised by PCE metabolites, resulting in passive leakage of Cr to urine [35]. From this, we can hypothesize that the non-significant result of creatinine from the above research could be due to the compensation secretion of creatinine by proximal tubules or due to leakage of Cr through compromised tubular epithelial barrier during the early stages of PCE Impact on kidney.

The urinary protein-to-creatinine ratio (UPCR) is a validated, spot-urine index that normalizes protein excretion to creatinine as a surrogate for glomerular filtration rate and urine concentration [36]. In clinical practice, spot urinary protein measurements are highly influenced by hydration status and urine volume, whereas creatinine is excreted at a relatively constant rate proportional to muscle mass, making it a reliable indicator of urine dilution [37]. In this study, both urinary protein and creatinine levels were significantly elevated in the dry cleaning group (P < 0.001 and P < 0.002, respectively); however, when expressed as a ratio, the effect of urine concentration is mathematically adjusted, resulting in a non-significant difference in UPCR (P = 0.791). This suggests that the higher absolute protein levels observed among dry cleaning workers likely reflect more concentrated urine rather than a true increase in renal protein excretion compared to hospitality workers. Similar with previous studies, absolute urinary protein levels may vary due to transient factors such as hydration and physical activity, whereas the UPCR provides a more stable and reliable indicator of renal function by accounting for urine concentration variability and enhancing diagnostic specificity by minimizing the confounding effects of variations in creatinine excretion [37,38].

Based on a few PCE-induced tubular damage studies, we formulated a scientifically relevant hypothesis related to urinary calcium and sodium and tested both of them. Urinary calcium is an important indicator of tubular function, which could detect early kidney damage caused by PCE, since the GSH metabolite results are believed to cause tubular toxicity [22]. Kidney reabsorbs calcium in order to balance body’s Ca level. But if anything happens to the kidney, such as being compromised by PCE exposure, hypercalcemia could be seen in urine [39]. As calcium output increases in urine, the chance of crystallization with other substances increases, leading to kidney stone or calcium phosphate formation [40]. Reabsorption of urinary calcium occurs in the proximal tubule, which would indicate a secondary change in ion handling as a result of PCE-induced toxicity, since GSH metabolites causes’ tubular damage. Urinary sodium would also show PCE toxicity in the same fashion as calcium, in addition to its ability to indicate electrolyte imbalance caused by the solvent [40–43].

Our research findings showed a median value of 2.6 mmol/l and 0.835 mmol/l for exposed and control groups, respectively. Just like our hypothesis, a higher value of calcium in urine output was seen in dry cleaners. The difference between the two groups was significant with p-value <0.001 for both independent t-test and Mann-Whitney test; however, dry cleaners account only 38.1% of the observed abnormal urinary calcium. Most of the abnormal results were below the normal range, which is less than 2.5 mmol/l. Most of the study participants have a calcium level below the normal range, but those who work in the dry cleaners have more urinary release as compared to hotel laundry workers.

Urinary sodium is an important biomarker to regulate the kidneys’ ability to maintain electrolyte balance. In normal conditions, 99% of Na is reabsorbed, where 70% of it is reabsorbed by the proximal tubule, 25% by the Loop of Henle in the thick ascending limb, and the rest 5% is absorbed by the distal tubule and collecting duct [44].

PCE metabolites could impair the thick ascending limb, which is a site for sodium reabsorption. This, in turn, results in disruption in the sodium-potassium-2-chloride (NKCC2) cotransporter, which results in increased urinary sodium [29], our study didn’t find any significant difference between the exposed and control groups.

Although no significant difference in BUN was found between the exposed and control groups, the data showed higher values in dry cleaners, though still within the normal range. A similar result was observed in a study conducted in Iran, where dry cleaners also exhibited higher BUN values within the normal range (17 mg/dl) than control groups (15 mg/dl) [21]. Most of the results are normal in our finding, but the higher value in dry cleaners indicates early renal stress caused by PCE exposure as a result early detection and routine monitoring would be valuable in order to prevent kidney toxicity, since long employment, PPE utilization and other factors could influence it even if we haven’t found significant correlation in our study for BUN.

### Limitation of the study

The study didn’t measure and quantify specific exposure assessment due to unavailability of direct reading devices and high cost of biological assessment, as a result, dose response linkage was not performed and since we used cross-sectional study, cause and effect relationship couldn’t be assured, by which the observed biomarker difference is unclear whether it was the result of cumulative exposure or recent exposure. Enzyme biomarkers such as like neutrophil gelatinase-associated lipocalin (NGAL) or kidney injury molecule-1 (KIM-1) were not assessed, due to substantially higher assay costs (often 5–10 times greater), limited commercial availability and specialized reagents/kits and we didn’t have the analytical machines for them in EPHI laboratory.

While PCE is the most likely causative agent based on exposure patterns, job tasks, and literature, residual confounding from co-exposures in dry cleaning cannot be fully ruled out without targeted environmental or biological monitoring of multiple solvents. We recommend that future studies in similar settings include multi-chemical exposure assessment (e.g., air sampling or urinary metabolites of other solvents) to further disentangle contributions.

## Conclusion

The finding showed a significant difference between dry cleaners and hotel laundry workers in TPU, urinary creatinine and calcium, which suggests PCE role on those biomarkers. Although the study failed to demonstrate a significant difference in UPCR, urinary sodium, BUN and serum creatinine, a higher prevalence of abnormality was seen in dry cleaners, which aligns with the biological plausibility of PCE-induced toxicity in dry cleaners. The cross-sectional nature of the study and the absence of direct exposure assessment hindered us from concluding definitively, but still the biomarkers showed a possible physiologic change and risk of nephrotoxicity as a result of the solvent., so our findings suggested that dry cleaners need to be more cautious as they are more susceptible to kidney toxicity however, a longitudinal study is needed to confirm the cause-and-effect relation.

## Supporting information

S1 Data Urinary raw dataset (SPSS format). Urinary biomarker data collected using ODK and exported to SPSS format.
(SAV)

S2 Data Serum raw dataset (SPSS format). Serum biomarker data collected using ODK and exported to SPSS format.
(SAV)

S3 Data Urinary raw dataset (CSV format). Urinary biomarker data collected using ODK and exported in comma-separated values (.csv) format; this file contains the same data as S1 Data.
(CSV)

S4 Data Serum raw dataset (CSV format). Serum biomarker data collected using ODK and exported in comma-separated values (.csv) format; this file contains the same data as S2 Data.
(CSV)

## References

[1] Agency for Toxic Substances and Disease Registry A. Public Health Statement for Tetrachloroethylene. Atlanta (GA): U.S. Department of Health and Human Services, Public Health Service. 2014. https://www.atsdr.cdc.gov/toxprofiles/tp18-c1-b.pdf

[2] European Chlorinated Solvent Association (ECSA). Health Profile PER December 2015. Brussels: ECSA. 2015. https://www.chlorinated-solvents.eu/wp-content/uploads/2015/12/Health-Profile-PER-December-2015.pdf

[3] Agency for Toxic Substances Disease Registry ATSDR. Toxicological Profile for Tetrachloroethylene. 2019. http://www.atsdr.cdc.gov/toxprofiles/tp18.pdf 37184172

[4] U.S. Environmental Protection Agency. Integrated Risk Information System (IRIS): Tetrachloroethylene (Perchloroethylene); CASRN 127-18-4. EPA. https://cfpub.epa.gov/ncea/iris/iris_documents/documents/subst/0106_summary.pdf. 2012. Accessed 2019 July 29.

[5] Salahudeen AK. Perchloroethylene-induced nephrotoxicity in dry-cleaning workers: Is there a role for free radicals?. Nephrology, Dialysis, Transplantation: Official Publication of the European Dialysis and Transplant Association-European Renal Association. 1998;13(5):1122–4. 9623539 10.1093/ndt/13.5.1122

[6] International Agency for Research on Cancer IARC. IARC monographs on the evaluation of carcinogenic risks to humans. 2014. https://monographs.iarc.fr/wp-content/uploads/2018/06/mono106-002.pdf PMC7681469 1683674

[7] Gobba F, Righi E, Fantuzzi G, Roccatto L, Predieri G, Aggazzotti G. Perchloroethylene in alveolar air, blood, and urine as biologic indices of low-level exposure. J Occup Environ Med. 2003;45(11):1152–7. doi: 10.1097/01.jom.0000094990.52172.ea 14610396

[8] Nakpan W. Occupational exposure of trichloroethylene: Toxicity, current standards and suggested new biomarkers for kidney cancer. PSRU Journal of Science and Technology. 2020;5(1):1–2.

[9] U.S. Environmental Protection Agency. Tetrachloroethylene (Perchloroethylene). EPA. https://www.epa.gov/sites/default/files/2016%2009/documents/tetrachloroethylene.pdf. 2016. Accessed 2024 August 15.

[10] Crews D, et al. Burden, access, and disparities in kidney disease. 2019. https://www.worldkidneyday.org/wp-content/uploads/2019/02/2019-KI-Final-Article.pdf 30851713

[11] Modenese A, Gioia TC, Chiesi A, Abbacchini C, Borsari L, Ferrari D, et al. Evaluation of occupational exposure to perchlorethylene in a group of italian dry cleaners using noninvasive exposure indices. Int J Environ Res Public Health. 2019;16(16):2832. doi: 10.3390/ijerph16162832 31398862 PMC6719957

[12] Guyton KZ, Hogan KA, Scott CS, Cooper GS, Bale AS, Kopylev L, et al. Human health effects of tetrachloroethylene: Key findings and scientific issues. Environ Health Perspect. 2014;122(4):325–34. doi: 10.1289/ehp.1307359 24531164 PMC3984230

[13] Cichocki JA, Guyton KZ, Guha N, Chiu WA, Rusyn I, Lash LH. Target organ metabolism, toxicity, and mechanisms of trichloroethylene and perchloroethylene: Key similarities, differences, and data gaps. J Pharmacol Exp Ther. 2016;359(1):110–23. doi: 10.1124/jpet.116.232629 27511820 PMC5034707

[14] Luo Y-S, Furuya S, Soldatov VY, Kosyk O, Yoo HS, Fukushima H, et al. Metabolism and toxicity of trichloroethylene and tetrachloroethylene in cytochrome P450 2E1 knockout and humanized transgenic mice. Toxicol Sci. 2018;164(2):489–500. doi: 10.1093/toxsci/kfy099 29897530 PMC6061689

[15] Brüning T, Bolt HM. Renal toxicity and carcinogenicity of trichloroethylene: Key results, mechanisms, and controversies. Crit Rev Toxicol. 2000;30(3):253–85. doi: 10.1080/10408440091159202 10852497

[16] Vyskocil A, Emminger S, Tejral J, Fiala Z, Ettlerova E, Cermanová A. Study on kidney function in female workers exposed to perchlorethylene. Hum Exp Toxicol. 1990;9(6):377–80. doi: 10.1177/096032719000900603 2271228

[17] Solet D, Robins TG. Renal function in dry cleaning workers exposed to perchloroethylene. Am J Ind Med. 1991;20(5):601–14. doi: 10.1002/ajim.4700200504 1793103

[18] Choi YH, Kim N, Seo YS, Choi SJ, Yang JO, Lee E-Y, et al. ARF requiring hemodialysis after accidental perchloroethylene ingestion. Am J Kidney Dis. 2003;41(3):E11. doi: 10.1053/ajkd.2003.50138 12613004

[19] de Morais DG, Sanches TRC, Santinho MAR, Yada EY, Segura GC, Lowe D, et al. Urinary sodium excretion is low prior to acute kidney injury in patients in the intensive care unit. Front Nephrol. 2022;2:929743. doi: 10.3389/fneph.2022.929743 37675036 PMC10479577

[20] Blood urea nitrogen (BUN) test. Mayo Clinic. https://www.mayoclinic.org/tests-procedures/blood-urea-nitrogen/about/pac-20384821. Accessed 2025 March 18.

[21] Ghahri A, Dehghan MH, Seydi P, Mashayekhi S, Naderi Y, Seydi E. The perchloroethylene-induced toxicity in dry cleaning workers lymphocytes through induction of oxidative stress. J Biochem Mol Toxicol. 2022;36(4):e23000. doi: 10.1002/jbt.23000 35156261

[22] Lash LH, Putt DA, Huang P, Hueni SE, Parker JC. Modulation of hepatic and renal metabolism and toxicity of trichloroethylene and perchloroethylene by alterations in status of cytochrome P450 and glutathione. Toxicology. 2007;235(1–2):11–26. doi: 10.1016/j.tox.2007.03.001 17433522 PMC1976278

[23] Al-Naimi MS, Rasheed HA, Hussien NR, Al-Kuraishy HM, Al-Gareeb AI. Nephrotoxicity: Role and significance of renal biomarkers in the early detection of acute renal injury. J Adv Pharm Technol Res. 2019;10(3):95–9. doi: 10.4103/japtr.JAPTR_336_18 31334089 PMC6621352

[24] Verplanke AJ, Herber RF, de Jong G, Mulder PP, van der Laan G. Occupational exposure to perchloroethylene and renal function. Occup Environ Med. 1999;56(6):386–90.

[25] Mutti A, Alinovi R, Bergamaschi E, Biagini C, Cavazzini S, Franchini I, et al. Nephropathies and exposure to perchloroethylene in dry-cleaners. The Lancet. 1992;340(8813):189–93. doi: 10.1016/0140-6736(92)90463-d 1353133

[26] Verplanke AJ, Leummens MH, Herber RF. Occupational exposure to tetrachloroethene and its effects on the kidneys. J Occup Environ Med. 1999;41(1):11–6. doi: 10.1097/00043764-199901000-00003 9924715

[27] Tang M. Glomerulus function in maintaining kidney health. Int J Nephrol Renovasc Dis. 2014;7:123–35.24729724

[28] Wedeen RP, Udasin I, Fiedler N, D’Haese P, De Broe M, Gelpi E, et al. Urinary biomarkers as indicators of renal disease. Ren Fail. 1999;21(3–4):241–9. doi: 10.3109/08860229909085086 10416201

[29] Dalaijamts C, Cichocki JA, Luo Y-S, Rusyn I, Chiu WA. Incorporation of the glutathione conjugation pathway in an updated physiologically-based pharmacokinetic model for perchloroethylene in mice. Toxicol Appl Pharmacol. 2018;352:142–52. doi: 10.1016/j.taap.2018.05.033 29857080 PMC6051410

[30] Tynkevich E, Flamant M, Haymann J-P, Metzger M, Thervet E, Boffa J-J, et al. Decrease in urinary creatinine excretion in early stage chronic kidney disease. PLoS One. 2014;9(11):e111949. doi: 10.1371/journal.pone.0111949 25401694 PMC4234219

[31] Stevens LA, Coresh J, Greene T, Levey AS. Assessing kidney function--measured and estimated glomerular filtration rate. N Engl J Med. 2006;354(23):2473–83. doi: 10.1056/NEJMra054415 16760447

[32] Vaidya VS, Ferguson MA, Bonventre JV. Biomarkers of acute kidney injury. Annu Rev Pharmacol Toxicol. 2008;48:463–93. doi: 10.1146/annurev.pharmtox.48.113006.094615 17937594 PMC2742480

[33] Cai SX, Huang MY, Chen Z, Liu YT, Jin C, Watanabe T, et al. Subjective symptom increase among dry-cleaning workers exposed to tetrachloroethylene vapor. Ind Health. 1991;29(3):111–21. doi: 10.2486/indhealth.29.111 1765547

[34] Andrea T, Isabella M, Rui F, Carrieri M, Bartolucci GB, Manno M. Kidney and liver biomarkers in female dry-cleaning workers exposed to perchloroethylene. Biomarkers. 2000;5(6):399–409. doi: 10.1080/135475000750052411 23898811

[35] Garimella PS, Tighiouart H, Sarnak MJ, Levey AS, Ix JH. Tubular secretion of creatinine and risk of kidney failure: The Modification of Diet in Renal Disease (MDRD) Study. American Journal of Kidney Diseases. 2021;77(6):992–4. doi: 10.1053/j.ajkd.2020.09.017 33221368 PMC8134514

[36] Guy M, Borrelli S, Kirkpatrick S, Major H, Parker T, Turner N. Evaluation of spot urine protein-creatinine ratio as a screening test for proteinuria. Ann Clin Biochem. 2009;46(1):20–5. doi: 10.1258/acb.2008.008134

[37] Stefańska K, Zieliński M, Zamkowska D, Adamski P, Jassem-Bobowicz J, Piekarska K, et al. Comparisons of dipstick test, urine protein-to-creatine ratio, and total protein measurement for the diagnosis of preeclampsia. Int J Environ Res Public Health. 2020;17(12):4195. doi: 10.3390/ijerph17124195 32545523 PMC7344421

[38] Ye Q, Shang S-Q, Liu A-M, Zhang T, Shen H-Q, Chen X-J, et al. 24h urinary protein levels and urine protein/creatinine ratios could probably forecast the pathological classification of HSPN. PLoS One. 2015;10(5):e0127767. doi: 10.1371/journal.pone.0127767 25996387 PMC4440756

[39] Audran M, Legrand E. Hypercalciuria. Joint Bone Spine. 2000;67(6):509–15. 11195313 10.1016/s1297-319x(00)00207-4

[40] Kim SW, Jeon JH, Choi YK, Lee WK, Hwang IR, Kim JG, et al. Association of urinary sodium/creatinine ratio with bone mineral density in postmenopausal women: KNHANES 2008-2011. Journal Title Abbreviation. 2011. 10.1007/s12020-015-0532-y 25614039

[41] Lauwerys R, Herbrand J, Buchet JP, Bernard A, Gaussin J. Health surveillance of workers exposed to tetrachloroethylene in dry-cleaning shops. Int Arch Occup Environ Health. 1983;52(1):69–77. doi: 10.1007/BF00380609 6874093

[42] Marouf BH. Health hazards, hematological and biochemical alterations in dry-cleaning workers using perchloroethylene. IJPS. 2022;31(2):144–9. doi: 10.31351/vol31iss2pp144-149

[43] University of California San Francisco (UCSF) Health. Medical Tests. https://www.ucsfhealth.org/medical-tests. Accessed 2024 October 10.

[44] Rose BD, Post TW. Clinical physiology of acid-base and electrolyte disorders. 5th ed. New York: McGraw-Hill. 2001.

